# Microscale Flow Control and Droplet Generation Using Arduino-Based Pneumatically-Controlled Microfluidic Device

**DOI:** 10.3390/bios14100469

**Published:** 2024-09-30

**Authors:** Woohyun Park, Se-woon Choe, Minseok Kim

**Affiliations:** 1 Department of Mechanical System Engineering, Kumoh National Institute of Technology, Gumi 39177, Republic of Korea; 2 Department of Aeronautics, Mechanical and Electronic Convergence Engineering, Kumoh National Institute of Technology, Gumi 39177, Republic of Korea; 3 Department of Medical IT Convergence Engineering, Kumoh National Institute of Technology, Gumi 39177, Republic of Korea; 4 Department of IT Convergence Engineering, Kumoh National Institute of Technology, Gumi 39253, Republic of Korea

**Keywords:** microfluidic device, flow control, solenoid valves, Arduino Uno, pneumatic pump, micro-droplet

## Abstract

Microfluidics are crucial for managing small-volume analytical solutions for various applications, such as disease diagnostics, drug efficacy testing, chemical analysis, and water quality monitoring. The precise control of flow control devices can generate diverse flow patterns using pneumatic control with solenoid valves and a microcontroller. This system enables the active modulation of the pneumatic pressure through Arduino programming of the solenoid valves connected to the pressure source. Additionally, the incorporation of solenoid valve sets allows for multichannel control, enabling simultaneous creation and manipulation of various microflows at a low cost. The proposed microfluidic flow controller facilitates accurate flow regulation, especially through periodic flow modulation beneficial for droplet generation and continuous production of microdroplets of different sizes. Overall, we expect the proposed microfluidic flow controller to drive innovative advancements in technology and medicine owing to its engineering precision and versatility.

## 1. Introduction

A lab-on-a-chip (LOC) refers to a microanalysis device designed to facilitate processes such as sample injection [1], pretreatment [2], chemical reaction [3], and separation/analysis [4] within microfluidic channels, enabling the analysis of chemical and biochemical substances at the laboratory scale [5]. A LOC exhibits precise capabilities in transporting [6], distributing [7], and mixing samples [8] with volumes as small as tens of microliters, allowing for rapid and straightforward analysis of chemical composition using minute amounts of samples. The recent emphasis on LOC applications underscores the need for microfluidic control systems [9,10] that facilitate various and selective sample injections, enabling the selective injection of diverse samples at the microscale. The microfluidic control system serves as a controller capable of delivering the desired flow rates into microchannels, aiding in the development of LOCs by facilitating tasks such as generating microdroplets [11], manipulating cells [12], detecting chemicals and biomolecules [13], and processing samples [14]. While microfluidic controllers capable of simultaneously providing flow rates for multiple fluids exist [10,15], hardware suitable for microscale flow rates is prohibitively expensive, making their deployment in small laboratory setups challenging [16]. Therefore, syringe pumps are widely employed for fluid flow control in small laboratory settings [17,18]. They allow users to define specific flow rates and are commonly used as substitutes owing to their ability to apply appropriate pressure drops to the microchannels.

While syringe pumps serve as suitable alternatives, they have disadvantages such as limitations in injection volume owing to structural constraints [19], pulsatile fluid flow [20], and a lack of understanding of the pressure exerted within microfluidic channels. In addition, channels employing multiple injection ports require multiple syringe pumps, further complicating the setup. To address these challenges, a novel flow control method utilizing consistent pressure and channel geometry was developed [21]. This method involves a pressure-regulated system in which users define the specific pressure and channel geometries to determine the flow rates. However, this approach cannot precisely determine the flow velocity and faces challenges in control owing to variations in the relationship between the flow rate and pressure depending on the channel geometry [22,23,24].

This study introduces a controller capable of creating precise and cost-effective microflows in laboratories conducting microfluidic experiments to alleviate the issues associated with syringe pumps. The proposed controller utilizes a system for pressure discharge and methods for modifying channel geometry to control flow rates. Moreover, the controller enables the independent adjustment of flow rates from multiple microfluidic inlet ports, allowing for the reproduction of various flow patterns. This study describes the fabrication and operation methods of a microfluidic controller, which predicts the flow rates based on the flow and pressure influenced by the microfluidic channel geometry, graphically represents the relationship and determines the ratio of the incoming flow rates based on the predicted values. Although fluid ratio adjustment and blocking can be achieved using discharge systems, rapid pressure differentials can lead to fluid backflow. To address this issue, this study proposes methods involving adjustments to the channel lengths at the inlet ports and using miniature regulators to mitigate abrupt pressure discharge. Finally, we demonstrate the functionality of the microfluidic controller essential for generating microdroplets of varying sizes that require fluid control and evaluate the performance of the developed microfluidic controller.

## 2. Experimental

### 2.1. Flow Control in Microfluidic Systems

Microfluidic flow dynamics can be broadly categorized into two main types: those driven by the intrinsic properties of fluids and those influenced by external forces [25]. Intrinsic properties of fluids include gravity [26], density [27], viscosity [28,29], and surface tension [30], and these generate forces due to the interaction between the microfluidic channels and the fluids at the microscale. These intrinsic properties play a crucial role in determining the flow behavior within microfluidic systems. Additionally, external forces such as electric fields [31], magnetic fields [32], and thermal fields, though relatively small in magnitude, can significantly impact and control micro-scale flow dynamics. By leveraging these external forces, precise manipulation and control of the fluid flow in microfluidic devices can be achieved, enabling various applications in fields such as lab-on-a-chip technologies and micro-scale chemical reactions. A common approach to drive microfluidic flow involves using surface tension, referred to as pressure-driven flow. In a pressure-driven flow, pressure is applied at the inlet of a microfluidic channel filled with fluid, which creates a pressure gradient along the channel length to propel the fluid movement. The volumetric flow rate of the fluid can be controlled using either a constant pressure or constant flow rate, with syringe pumps considered a typical example. When the hydraulic resistance of the microfluidic channel doubles, the syringe pumps automatically increase the pressure necessary to achieve the desired flow rate. This approach, independent of channel structure, is highly beneficial for various microfluidic applications. However, syringe pumps are prone to flow oscillations at low flow rates (~0.1 μL/min) [33], leading to microscale inaccuracies. One method to address this issue involves applying a fixed pressure to the microchannels to induce net flow and generate flow using constant pressure, which offers advantages such as rapid response times, high stability, pulseless flow, and the ability to handle large fluid volumes [34]. However, precise control of flow rates may pose challenges in applications that require exact control. Nevertheless, pressure–flow rate curves for specific microfluidic devices can be obtained by capturing the fluid at the outlet of the microfluidic channel and calculating the collected volume over time as a function of various pressure values. Ultimately, pressure-driven systems enable precise flow rate control [35,36].

### 2.2. Arduino-Based Flow Controller Manufacturing Method and Appearance

**Figure 1:**
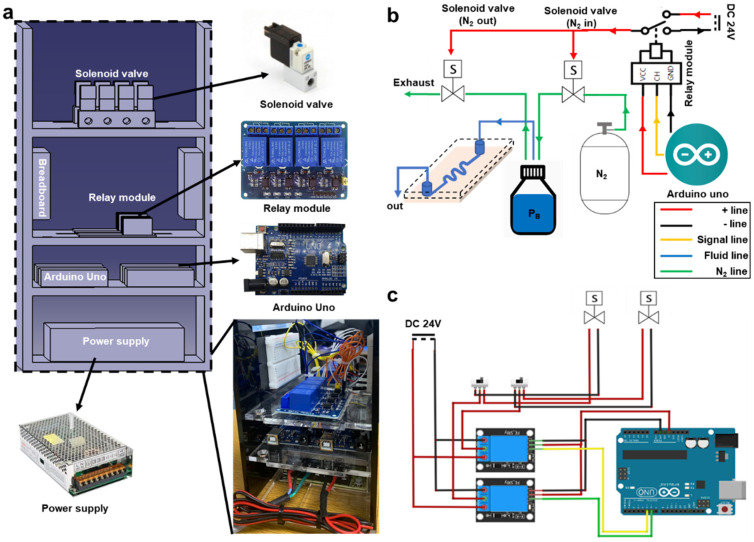
Microfluidic control system. (**a**) Shape of constructed microfluidic control system and appearance of components used. (**b**) Schematic diagram of constructed microfluidic control system. (**c**) Electrical circuit diagram of constructed microfluidic control system.

The pneumatic microfluidic controller utilized in this study comprised one Arduino Uno, one relay module, two solenoid valves, and one power supply unit. Commercially available pneumatic flow controllers are typically priced around USD 15,000. In contrast, the pneumatic microfluidic flow controller developed in this study can be produced at approximately USD 150, making it about 1/100th the cost of existing products (Table 1). The Arduino Uno served as the command system for the pneumatic microfluidic controller, whereas the power supply unit provided the necessary voltage for the solenoid valve operation. The relay module facilitates voltage separation between the power supply unit and Arduino Uno. The pneumatic flow controller fabricated in this study was constructed using acrylic material and comprised four layers. An Arduino Uno, two solenoid valves, and two relay modules were required to control a single flow. The pneumatic flow controller developed in this study can regulate flows, with additional flows easily accommodated by adding components: the power supply occupies the first layer, the Arduino Uno is positioned on the second layer, the relay module is situated on the third layer, and the solenoid valve is located in the fourth layer. This configuration was devised to minimize wire pathways and flow obstructions (Figure 1a).

### 2.3. Microfluidic Control Method Employing Pneumatic System

The development of the microfluidic controller in this study was initiated by introducing nitrogen gas into a bottle containing distilled water through two solenoid valves (V200; Shinyoung Controller, Seoul, Republic of Korea). The pressure in each bottle was adjustable. To increase the internal pressure of the bottle, the solenoid valve (inlet valve) connected to the nitrogen gas tank was opened, whereas the solenoid valve (outlet valve) connected to the atmospheric pressure was closed. Conversely, if the solenoid valve connected to the nitrogen gas tank was closed and the solenoid valve connected to the atmospheric pressure was opened, the nitrogen gas inside the bottle was released to atmospheric pressure, resulting in a pressure decrease. A movable pipe connected to distilled water was used during this process. Subsequently, the nitrogen gas remaining inside the bottle pushed the distilled water. When the expelled distilled water is directed into a microfluidic channel with microscale dimensions, a microflow can be established. In conclusion, the pressure within the bottle could be adjusted by regulating the nitrogen gas remaining inside the bottle, thereby regulating the flow rate and volume of distilled water entering the microfluidic channel (Figure 1b). In this regard, there may be potential concerns about the impact of gas compressibility on droplet formation under high-flow conditions in pneumatic-driven systems. However, in the microfluidic flow controller developed in this study, gas compressibility does not significantly affect the flow rate. This is because the flow rate in the microfluidic system is very small, approximately 50 μL per hour. The small volume of displaced fluid minimizes the effect of gas compressibility on the flow rate entering the microfluidic channels. Additionally, the pneumatic system uses a reservoir with a relatively large capacity of 500 mL compared to the flow rate, further reducing the influence of gas compressibility. Moreover, nitrogen gas, which has low reactivity, is used to minimize variations in flow rate due to gas compressibility. For these reasons, the pneumatic flow controller developed in this study effectively minimizes the impact of gas compressibility on the flow rate.

### 2.4. Solenoid Valve Control System

The microfluidic controller described in this paper was controlled using an Arduino command system, which enables configuring parameters such as the open duration, closed duration, and solenoid valve cycle. However, operating the solenoid valve required a power supply of 24 V, exceeding the Arduino Uno voltage limit of 15 V. Consequently, applying an external 24 V power source to operate the solenoid valve risks damaging the Arduino Uno circuitry. This limitation can be circumvented using a relay module. With the relay module, the control system circuit and power supply circuit can be separated, thus preventing excessive voltage from reaching the Arduino Uno. The relay module receives commands from the Arduino Uno to activate or deactivate the power supplied to the solenoid valve, thereby enabling control over the operating duration and cycle (Figure 1c).

### 2.5. Fabrication of Master Mold

The microfluidic device was fabricated by standard photolithography. To create the layers, a negative photoresist (SU-8 2025; MicroChem, Newton, MA, USA) was spin-coated onto a silicon wafer (Unisill, Seoul, Republic of Korea), soft-baked to form a layer and then exposed to UV light using a photomask (Microtech, Bucheon-si, Gyenggi-do, Republic of Korea) and a mask aligner (MDA-60MS, MidasSystem, Daejeon, Republic of Korea). Dipping the wafer into a Su-8 developer (Kayaku Advanced Materials, Westborough, MA, USA) removed the unexposed photoresist and completed the layered master mold. Subsequently, the surface of the master mold was coated with trichloro(3,3,3-trifluoropropyl) silane (Sigma-Aldrich, Seoul, Republic of Korea) in a vacuum jar for 1 h. Subsequently, the PDMS solution was degassed in a vacuum jar, cast, and cured at 60 °C for 4 h. The PDMS channel and glass slide were directly bonded via an oxygen plasma treatment (Cute-MP, Femto Science, Hwaseong-si, Gyeonggi-do, Republic of Korea) at 50 sccm O_2_ and 50 W for 30 s. To prevent contamination, each microfluidic device used in this study was used only once in each experiment

### 2.6. Experimental Setup and Data Analysis

A digital fluorescence microscope (F1-CIS; Nanoscope Systems, Daejeon, Republic of Korea) equipped with a charge-coupled device camera and lens was used to acquire a bright field. The flow measurement involved collecting the fluid passing through the resistance microfluidic device for a specific duration and using a scale to measure its weight. The ImageJ 1.x software (NH, Bethesda, MD, USA) was used to measure the ratio of the different fluids occupying the microfluidic device. Subsequently, various data analyses, including graphing, were performed using Origin 9.0 (OriginLab, Northampton, NC, USA).

## 3. Result and Discussion

### 3.1. Analysis of Flow Rate Dependence on Pressure Microfluidic Channel Length

**Figure 2:**
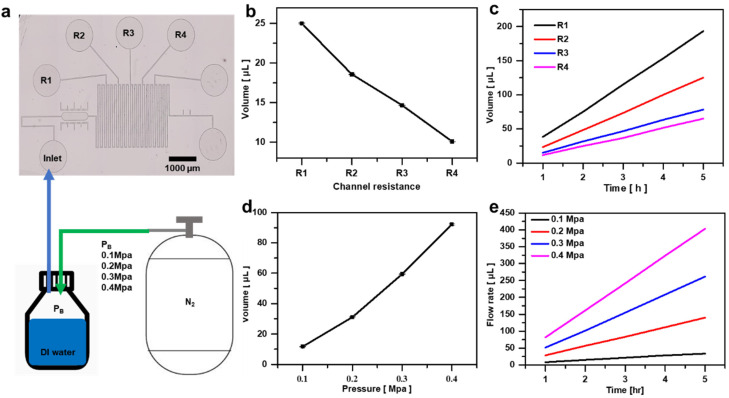
Flow measurement of the flow control system. (**a**) Schematic illustrating the method of increasing the pressure in the syringe to introduce fluid into the microchannel. (**b**) Graph depicting the flow rate measured over 1 h with syringe pressure fixed at 0.2 MPa and varying channel lengths. (**c**) Graph illustrating the flow rate measured at 1 h intervals over 5 h with syringe pressure fixed at 0.2 MPa and varying channel lengths. (**d**) Graph depicting the flow rate measured over 1 h with the channel length fixed at R1 and varying syringe pressure. (**e**) Graph illustrating flow rates measured at 1 h intervals over 5 h with the channel length fixed at R1 and varying syringe pressure.

Syringe pumps can maintain a fixed flow rate, but they have limitations in accuracy due to minor rotational errors. To overcome these limitations, this study proposes a microfluidic flow control system that ensures high precision. This system is based on constant pressure, where the flow rate varies depending on the pressure and the length of the channel. As the pressure in the bottle increases, the flow rate also increases, and the flow rate changes based on the channel length due to the friction (flow resistance) with the channel walls as the fluid passes through the microfluidic channel. When the cross-sectional area of the channel decreases, the resistance increases, requiring higher pressure to maintain the same flow rate. This phenomenon is explained by Poiseuille’s law, which shows that, as the channel narrows, the pressure drop becomes greater. Therefore, to precisely control the flow rate in a constant pressure system, it is necessary to consider the changes in flow rate according to channel length and pressure. To derive the flow rate curve in a microfluidic device, fluid must be collected at the outlet, and the volume collected over time is measured under varying pressure and channel lengths to calculate the flow rate. This method allows the desired flow rate to be set accurately (Figure 2a).

Initially, to assess the flow-rate variation with the channel length, nitrogen gas pressure was held constant at 0.2 MPa, with the flow rate measured as the channel length changed, as illustrated in a flow rate diagram (Figure 2b). Subsequently, to evaluate whether the flow rate remained consistent with the changes in the nitrogen gas length over an extended duration, the nitrogen gas pressure was fixed at 0.2 MPa, while the channel length was altered; the flow rate diagram was recorded for 5 h (Figure 2c). Analysis of the flow rate curve in Figure 2a,b reveals a linear trend for the flow rate, indicating a proportional relationship between the channel length and the flow rate. In addition, measuring the flow rate in response to pressure changes indicated a linear increase in the flow rate with increasing pressure within the bottle, demonstrating the flow-rate variation corresponding to pressure changes further illustrated by the flow rate curve based on pressure (Figure 2d). Furthermore, to verify the sustained flow rate over an extended period with pressure fluctuations, the channel length was fixed at R1, and the pressure variations were recorded for 5 h. The resulting flow rate diagram depicted a consistent linear graph, confirming the continuous maintenance of a constant flow rate (Figure 2e). In summary, the flow rate exhibited dependencies, as depicted in the graphs, and was primarily influenced by both the channel length and the pressure within the bottle. The flow rate curves provided insights into the flow-rate variations with respect to the channel length and pressure fluctuations within the bottle.

### 3.2. Pneumatic Discharge Method for Flow Control

**Figure 3:**
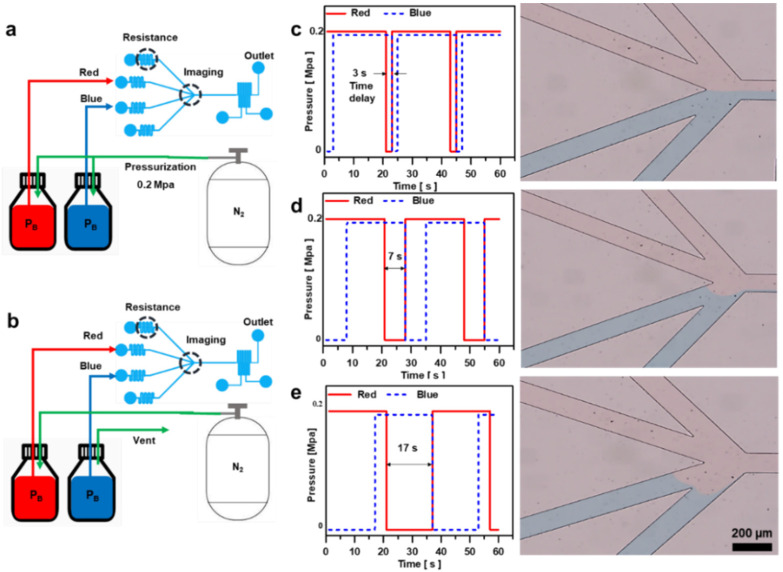
Microchannel geometry and flow control system utilizing the feedback system of Arduino Uno. (**a**) Schematic of the fluid infusion method. (**b**) Schematic of the fluid blocking method. Discharging the pressure of the bottle containing the fluid that blocks the inflow to atmospheric pressure, maintained for (**c**) 3 s, (**d**) 7 s, and (**e**) 17 s.

Controlling a single flow is inadequate in many microfluidic applications. For instance, cell biology experiments often require the simultaneous flow of multiple fluids, demanding a broader spectrum of microfluidic capabilities. Therefore, a suitable fluid-flow system is required. In this study, we present a simple and cost-effective flow control method, which regulates the pressure by releasing nitrogen gas into the bottle until it reaches atmospheric pressure, thereby controlling the volume of fluid entering the channel. The procedure is as follows: Initially, when maintaining identical pressure in bottles containing blue-dyed distilled water (BD) and red-dyed distilled water (RD), equal volumes of BD and RD flow into the microfluidic channel (Figure 3a). Subsequently, upon releasing nitrogen gas at atmospheric pressure into the bottle of the designated fluid, a pressure differential arises between the fluids, resulting in disparate flow rates entering the microfluidic channel (Figure 3b). Due to sudden pressure changes, there is a possibility that the fluid deviates from the intended outlet path and moves toward the bottle in a low-pressure state, leading to the mixing of the two fluids. To mitigate this issue, we extended the length of the microfluidic channel at each fluid inlet to introduce resistance to the flow, thereby minimizing backflow. However, this extension of the channel length resulted in a decrease in the reaction speed due to pressure. By appropriately adjusting the length of the channels at the inlets, we were able to maintain precise flow control while simultaneously reducing the response time to pressure changes.

The flow rate entering the microfluidic channel can be regulated by adjusting the discharge time of nitrogen gas in the bottles containing RD and BD visually observed using an optical microscope to validate the feasibility of the flow-rate control. As the discharge time of nitrogen gas increased, the area occupied by the fluid in the channel gradually decreased. Consequently, flow rate control and blockage can be achieved by modulating the nitrogen gas discharge time. Furthermore, employing the Arduino loop statement enabled periodic flow-rate control, confirming the potential for both flow-rate regulation and interception (Figure 3c–e).

### 3.3. Multiple Flow Rate Control Utilizing the Pneumatic Discharge Method

**Figure 4:**
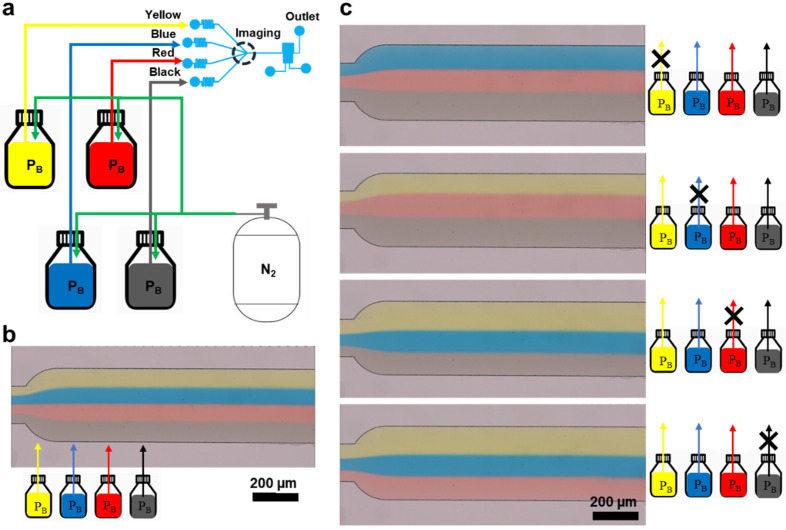
Adjustment of the flow of multiple fluids using the microchannel geometry and Arduino Uno’s feedback system. (**a**) Schematic of the method for controlling the flow of four different fluids using Arduino Uno’s feedback system. (**b**) Digital fluorescence microscope image capturing the area occupied by the fluid in the channel applying the same pressure to each fluid. (**c**) Digital fluorescence microscope image depicting a single fluid obstruction occupying a portion of the channel.

Microfluidic applications require a broad spectrum of fluid flows. To validate this aspect, we aimed to expand the range of fluids and assess the efficacy of flow control and blocking. We used distilled water that was dyed yellow (YD), black (BLD), red (RD), and blue (BD). The experimental approach involved inducing a pressure differential within a fluid-containing bottle by venting nitrogen gas outside the bottle. An experiment was conducted using this methodology (Figure 4a).

Initially, we regulated the amount of nitrogen gas in each fluid-containing bottle and observed the area occupied by each fluid in the channel under a microscope. Upon applying equal pressure to bottles containing different fluids, we observed a 1:1:1:1 ratio of the channel area occupation, suggesting that, when the number of fluid molecules is increased while maintaining a consistent pressure, an equivalent volume flows into the microfluidic channel (Figure 4b). In addition, we examined the flow of individual fluid types under a microscope to assess the feasibility of interception. Upon releasing nitrogen gas from a bottle containing the targeted fluid, the internal pressure decreases, creating a pressure gradient with other fluid-containing bottles. Consequently, the reduced pressure hindered the flow of fluid from the bottle into the channel, resulting in the occupation of the channel space by the three fluids (Figure 4c). This result demonstrates the possibility of maintaining a constant flow rate and selectively blocking it even with an increased variety of fluid types, further confirming the feasibility of sustaining and obstructing the flow rates for various fluid types.

### 3.4. Pressure Regulation Utilizing a Pressure Regulator

**Figure 5:**
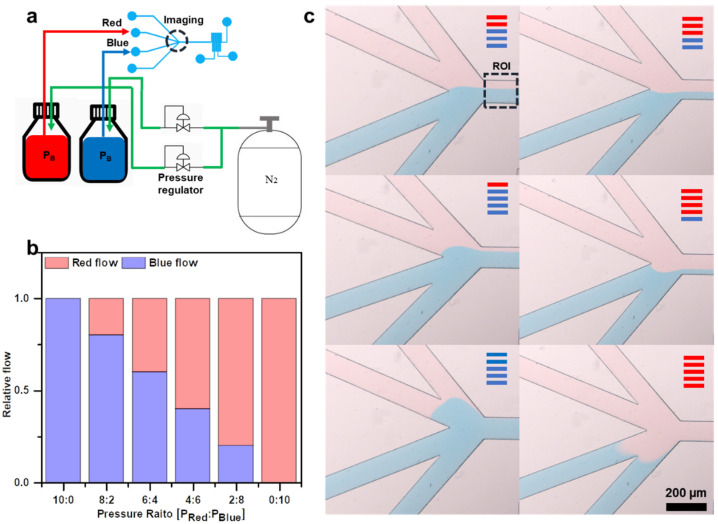
Method of adjusting the flow entering each bottle using a regulator. (**a**) Schematic illustrating the method of regulating the flow entering the channel using a regulator. Area occupied by the fluid in the channel adjusting the pressure inside the bottle using a regulator (**b**) graph. (**c**) Digital fluorescence microscope picture.

The approach of regulating the pressure by venting nitrogen gas from the bottle to atmospheric pressure and controlling the flow rate into the microfluidic channel offers the advantage of repeated flow-rate adjustments. However, this method suffers from the backflows of time-consuming adjustments and flow rate interception. To address these limitations, we propose a method for regulating the flow rate into the microfluidic channel by controlling the inflow of nitrogen gas into the bottle using a pressure regulator. This method involves generating a pressure differential by varying the amount of nitrogen gas inflow rather than relying on nitrogen gas discharge. Adjusting the nitrogen gas inflow prevents sudden pressure differentials, thereby eliminating the backflow phenomenon and eliminating the need for additional channel-length adjustments (Figure 5a).

By employing a regulator within the bottles containing the RD and BD, the inflow of nitrogen gas could be regulated and assessed through microscopic examination. The varying inflow amounts of nitrogen gas resulted in differential pressures being applied to each fluid, thereby influencing the microfluidic channel proportions (Figure 5b,c). In summary, this method offers the ability to control flow rates, block flows, and achieve rapid response times by adjusting the nitrogen gas inflow. However, this approach entails manual control and does not support automatic flow rate adjustments. Nevertheless, this approach is suitable for experiments characterized by extended dwell times and scenarios wherein fluid movement must be maintained at a constant rate.

### 3.5. Micro-Droplet Fabrication Utilizing a Pressure Regulator

Droplet-based microfluidic systems have garnered significant interest owing to their potential applications in material synthesis, drug encapsulation, and protein crystallization. These systems operate by separating immiscible fluids, such as water and oil, using microfluidic channel interfaces and precisely controlled flow rates. The underlying principle involves guiding water through a microfluidic channel where its movement is constrained and segmented into droplets by a continuous flow of oil (an immiscible fluid) along the channel. Droplet formation occurs at the interface between the separated fluid (distilled water) and continuous oil flow. Various microscale fluids can be separated based on the flow rate between fluids (oil). However, achieving this separation requires accurate flow rate control. Therefore, we propose a droplet-separation system that utilizes the microfluidic controller developed in this study.

**Figure 6:**
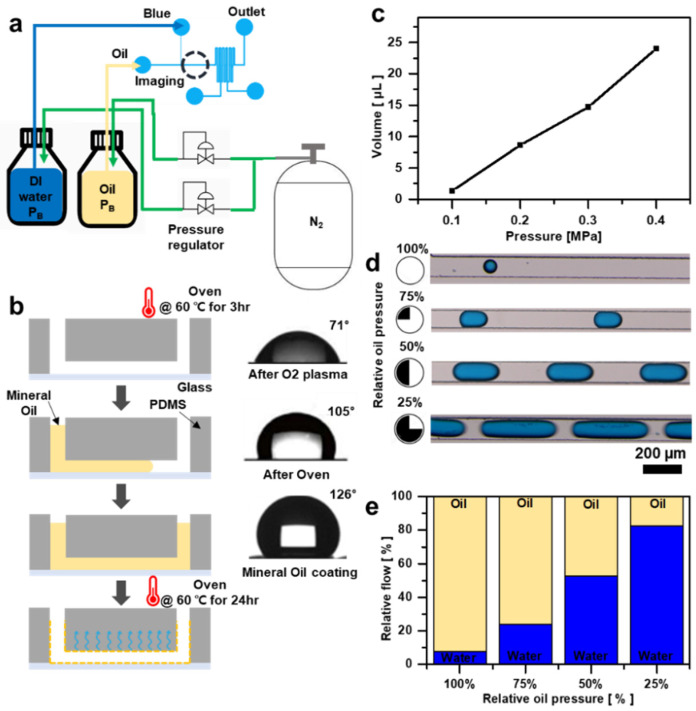
Generating microfluidic droplets using DI water and mineral oil. (**a**) Schematic depicting the method of generating microfluidic droplets using microchannel geometry and a regulator. (**b**) Surface processing procedure of the microchannel. (**c**) Graph illustrating the flow rate of mineral oil in the microchannel at different pressures. (**d**) Digital fluorescence microscope image depicting variation in droplet size as the flow rate of mineral oil is adjusted to 100%, 75%, 50%, and 25%. (**e**) Graph illustrating the ratio of DI water and mineral oil occupying the channel as the flow rate of oil is adjusted.

To maintain a constant flow rate for continuous operation, a pressure regulator was used to manage the pressure inside the bottle by regulating the flow of nitrogen gas. Blue-dyed distilled water and mineral oil were used as the fluids in this experiment. To enhance the shear force between the fluids within the channels, the T-junction method was adopted, which allowed for vertical contact between the two fluids (Figure 6a). However, relying solely on the shape of the channel to implement a droplet system is challenging. The channel to implement a droplet system is challenging. The channel interface plays a crucial role in determining the success of droplet separation. Therefore, an additional step is necessary to render a microchannel interface made of superhydrophobic PDMS to facilitate smooth droplet separation. Although PDMS is inherently hydrophobic, the O_2_ plasma process used to bond the glass slide to the microfluidic channel renders the channel interface hydrophilic, resulting in a contact angle of approximately 71 degrees. To address this issue, the microfluidic channel’s interface is restored to a hydrophobic state by heating it in an oven at 60 °C, which increases the contact angle to about 105 degrees. Subsequently, a mineral oil coating was applied involving injecting mineral oil into the microfluidic channel and heating it in an oven at 60 °C for 24 h to achieve super-hydrophobicity. This procedure presses the mineral oil onto the microfluidic channel interface, ultimately creating a superhydrophobic state with a contact angle of approximately 126 degrees (Figure 6b).

Hence, microscale flow-rate separation can be achieved by employing the mineral oil coating method on the T-junction microfluidic channel. The separation of the flow rates depends on the ratio of distilled water to mineral oil. In this study, analyzing the flow rate of mineral oil is essential to set the appropriate flow rate for experimentation. The flow rate of mineral oil in the T-junction microfluidic channel was monitored for 1 h at pressures ranging from 0.1 to 0.4 MPa to select the required flow rate (Figure 6c). Subsequently, the inflow of distilled water remained unadjusted, whereas the inflow of mineral oil was controlled by regulating the pressure of nitrogen gas using a pressure regulator. An optical microscope was used to observe the cases in which 25%, 50%, 75%, and 100% mineral oil were applied at a pressure of 0.2 MPa. Consequently, the microdroplet size was adjusted by controlling the oil inflow (Figure 6d). Furthermore, an examination of the graph illustrating the ratio of mineral oil to distilled water in microfluidic channels of equal length shows that, as the flow rate of distilled water increases, the volume of the liquid also increases, leading to a reduction in the droplet size (Figure 6e). Given these observations, the microfluidic controller developed in this study demonstrates the capability to generate microdroplets of varying sizes, thereby offering potential applications across diverse fields.

## 4. Conclusions

Precise fluid flow control is essential in microfluidic applications, but existing commercial flow controllers and syringe pumps are costly and inefficient, particularly in the field of biology. In response, this study proposes a cost-effective, micro-scale flow controller optimized for microfluidic systems, offering an innovative approach in terms of cost savings and flexibility. However, like any scientific research, this study also has some limitations that could affect the results and conclusions. The first limitation relates to constraints in manufacturing and use. While the system utilizes a custom micro-scale flow controller to regulate fluid flow, optimal performance is only achieved under specific conditions. Unexpected flow control issues may arise depending on the environment. For example, pneumatic control methods may struggle to maintain predicted flow rates due to factors such as channel geometry or the physical properties of the fluid. Nevertheless, simple flow analysis can help maintain consistent flow rates, and adding a flow meter can further improve flow predictability. Although the primary goal of this study was to develop a low-cost pneumatic flow controller, integrating a flow meter would enable more precise control. A significant advantage of this system is that it addresses the issue of flow instability at low flow rates, a common problem in syringe pumps caused by motor vibrations. The second limitation concerns response time and backflow issues. The flow controller developed in this study adjusts the cross-sectional area of the inlet channel to reduce backflow. However, increasing the cross-sectional area can lead to longer response times in the system due to changes in pneumatic pressure. To improve response time, reducing the cross-sectional area may be effective. The system uses flow-pressure and resistance diagrams based on channel length to control the flow, providing predictable flow regardless of the microfluidic channel’s geometry. Additionally, adjusting the channel length or pressure allows for effective fluid control without undesirable backflow. This system facilitates droplet generation by manipulating the channel interface and flow, demonstrating its versatility in various microfluidic applications. It can generate micro-droplets of desired sizes using different flow control techniques. With its ease of assembly and operation, as well as its ability to predictably control and block fluid flow, the system offers significant advantages in terms of much lower manufacturing costs compared to a single syringe pump. Given these factors, the system is expected to enable fast, simple, and cost-efficient experiments in a wide range of microfluidic applications. Future research aimed at addressing these limitations and improving the system will be crucial for expanding its applicability.

## Figures and Tables

**Table 1:** Specifications of core components.

Part	Voltage	DC Current Per I/O Pin	Dimension	Cost
Arduino Uno	7~12 V	40 mA	68 × 53 mm^2^	~10 USD
Relay module	5 V	5 mA	43 × 17 × 18 mm^3^	~5 USD
Power supply	24 V	5 A	198 × 100 × 43 mm^3^	~60 USD
Solenoid valve	24 V	9 mA	10 × 40 × 18 mm^3^	~25 USD

## Data Availability

The original contributions presented in the study are included in the article, further inquiries can be directed to the corresponding authors.
